# Housekeeping Queries

**Published:** 1912-11-02

**Authors:** 


### INSTITUTIONAL HOUSEKEEPING.

### Housekeeping Queries.

**The Store-room Maid.**—It is quite true that the ideal store-rooms described in our issue of September 14 would necessitate more labour. In fact, the store-rooms cannot be properly organised in any large establishment unless the energies of one maid be devoted to the work. It is very usual to depend on help from the kitchen for service which the housekeeper requires in connection with the unpacking and distribution of the stores, and on one of the porters for the cleaning of floors, etc. It is, however, a far better plan to have a well-trained maid attached to the housekeeper as her aide-de-camp for all these offices, so that one trustworthy person is in charge. Floors need to be wiped over with great care lest dust be stirred on goods exposed to view. Shelves need to be kept in a spotless condition of cleanliness. Jars require constant washing as they are emptied and refilled. Knives must be kept polished. Labels need to be written. Long processes of measuring and weighing have to be gone through. There are many things, such as pickles, jams, furniture polish, etc., which could be economically made on the premises if the housekeeper had sufficient labour at her disposal. And should there be spare time a few hours weekly could be devoted to the work-room. We are sure that if you once tried the plan of having one girl to train for this special branch of work you would find your store-room maid indispensable.—Sister J.

**The Advantages of Lady Cooks.**—To specify all the ways in which a lady could prove her superiority would prove a lengthy task. Perhaps her best work would lie in organising the kitchen staff and training subordinates. If thoroughly efficient, she should be able to undertake the catering and take a great deal off the matron's shoulders. She would be housekeeper and cook in one, at any rate as regards the commissariat. Moreover, even in smaller establishments she would exercise an immense influence in introducing taste and refinement into the manner in which the food was sent to table, and this counts for a great deal when the consumers sit down tired and disinclined for their meal. For instance, the ordinary steamed or boiled "gigot" sent to table masked in a good parsley sauce and garnished with stars of carrots and turnips is wonderfully more attractive than the same article sent in straight from the pot. The lady cook takes a pleasure in its nice appearance. The very breakfast bacon can be manipulated into a dainty dish by using up pieces of cut bread left from supper the night before to fry and serve under or round it. The same refining influence ought to pervade every meal served in the establishment from the breakfast tray of the invalid nurse to the serving of the kitchen dinner. The pity of it is that there are so few lady cooks to be had.—Joice.

**Separate Tables for Meals.**—The objections you raise should have every consideration. It is undoubtedly inconvenient to have the room filled with small tables if it is to be used as a general sitting-room as well as dining-room, but this is a most objectionable arrangement where numbers are to be accommodated. Quite apart from the question of airiness and comfort, no nicely-laid meals can be expected when the parlourmaids cannot have free access to the tables for laying and clearing. A stronger objection may lie in the additional cost of laundry when in place of the regulation two long cloths a week some dozen or more have to be washed. On the other hand, small cloths are much handier to fold and cheaper to buy, while the laundress much dislikes the long, heavy table napery. Wherever the plan of serving meals on several small tables instead of one long one has been tried it has won its way, and has resulted in a more sociable and refined atmosphere, which helps to make meal times the real recreation they ought to be.—Sister Martha.

**The Inevitable Milk Pudding.**—Nothing, as you justly complain, is more monotonous than the ordinary milk pudding, and it is surprising it holds its own so long. Try the following variation: Take the sago, rice, or other cereal, and cook it gently first in milk, duly flavouring it with sugar. Then stir in the yolks only of the eggs with more milk, and let it just set in the oven. Before sending it to table beat up the whites with a spoonful of jam or with powdered sugar only; lay this on the top, place in the oven to set once more, and serve. It then becomes a delicacy to be desired, an excellent sago or rice custard, not merely good to look at, but more nourishing than the ordinary baked kind.—Ella.

**Vegetable Stock.**—No wonder your meatless soups are voted tasteless if you neglect to have *vegetable stock* prepared, it is as necessary as the meat stock which is prepared in every household. Make it in this way: Wash, prepare, and cut up one carrot, turnip, small beetroot, parsnip, and lettuce. Also two onions and a leek, if possible. Put all these into a saucepan with some outside sticks of celery and two or three cabbage leaves shredded. Pour in two quarts of boiling water, add a dozen sprigs of parsley, two bay leaves, three cloves, and one teaspoonful of mixed herbs; the two latter ingredients are best tied up in muslin. Add salt and pepper, and simmer until the vegetables are soft. Then strain off the stock through a fine sieve, and use it for the foundation of soups or sauces. Any other seasonable vegetables can be used, and, if liked, they can be rubbed through the sieve to thicken the stock.—Sister Norah.

**Inflammable Frying Pans.**—You are wise to have taken into consideration the risk from pans of frying fat taking fire, for many lives and much property have been lost through this cause. The best way will be to hang clearly-printed directions up in your kitchen as to what to do if the fat is allowed to over-heat and so burst into flame. (1) Fat from which smoke rises in a very thick cloud will soon catch fire unless cooled down. (2) If fat catches fire *never throw water* on it. (3) Put flour, salt, or sand gently in from a shovel, never throw them. (4) Try and slip on a lid to smother the flames. (5) Keep far away from the burning pan.—Nervous Housekeeper.

**Heavy Mattresses.**—Large mattresses are trying to turn about, but much easier if you have something to grip hold of. Try sewing on the sides or ends handles made of small straps, strong broad tape or strips of mattress ticking.—Single-handed.

**New Flannels.**—To remove the bluish tint from new Welsh flannels, dissolve some soap and let it cool till it becomes a jelly. Rub thoroughly every portion of the flannel over with this jelly. Leave for five minutes and then wash as for ordinary flannel.—Laundry Novice.

